# Self-Cleaning Textiles for Publicly Shared Soft Surfaces: An Accessible and Scalable Photocatalytic Polyester System with Embedded Light-Emitting Diodes

**DOI:** 10.1021/acsomega.4c09837

**Published:** 2025-01-24

**Authors:** Aditi Maheshwari, Fiona Bell, Eric Gallo, Mihai Ibanescu, Emily Robertson, Charlotte Fairless, Francis Logan, Mary Jane Schmuhl, Henry Cheung, Michael Rein, Andreea Danielescu, Mirela Alistar

**Affiliations:** † Accenture Labs, San Francisco, California 94105, United States; ‡ University of Colorado Boulder, Boulder, Colorado 80309, United States; § Advanced Functional Fabrics of America, Cambridge, Massachusetts 02139, United States

## Abstract

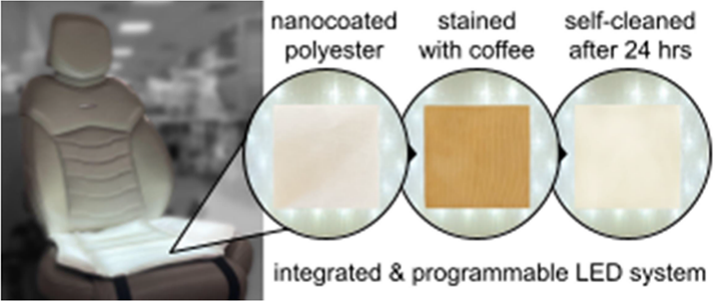

Shared textiles used in public spaces (transportation, theaters, etc.) are notoriously difficult and expensive to clean, resulting in infrequent cleanings. To address these challenges, we developed a light-diffusing textile system with embedded LEDs that can self-clean, i.e., degrade organic stains and inhibit bacterial growth. Our self-cleaning textile system can be triggered at any time, bypassing the need for additional chemicals and manual cleaning. Specifically, we tested commercial titanium dioxide nanoparticles doped with nitrogen on polyester, applied four different ways (painting, rolling, spraying, and dip coating) with two different curing methods (7 days at room temperature and 10 min at elevated temperatures) and different textile surface pretreatments (no pretreatment, corona discharge, commercial primer, and corona discharge with commercial primer), and compared their efficacies using stain degradation testing and EDS analysis to arrive at the best performing textile cover. We then integrated LED fibers into this cover to create a self-cleaning system capable of autonomously cleaning the textile on demand. We evaluated our system through stain degradation experiments and bacterial testing, demonstrating that stain degradation increases linearly with the duration of exposure and LED intensity. Our results verify that our textile system effectively destains the textile in both dark and bright ambient conditions within 2 days and inactivates both Gram-positive and Gram-negative bacteria up to 99% after 8 h of exposure. Finally, we stack LED fibers with reflective and diffusive textile layers for modularity and programmability. This self-cleaning system can be seamlessly integrated into public environments such as automotive or theater seatings, offering enhanced hygiene and cleanliness without compromising comfort.

## Introduction

High-touch textiles used in public spaces are difficult to clean regularly leading to such textiles accumulating stains and potentially pathogenic microbes.^1^ The CDC recommends using a combination of a fabric-appropriate cleaner and a vacuum to clean public textiles, however, the process of disinfecting public spaces thoroughly is time-consuming, labor-intensive, and requires additional chemicals (that can be environmentally toxic).^2^ For that reason, many public spaces (like buses, hospitals, etc.) traded comfort for cleanliness by replacing soft textiles with rigid plastic surfaces that can be easily disinfected.^3^ Such a trade-off is not ideal in spaces where the user experience is critical, such as in theaters, or commercial vehicles. To address these challenges, we developed a *self-cleaning textile system* that can be activated by light-emitting diode (LED) fibers, bypassing the need for additional chemicals and manual cleaning. Our contribution lies in integrating programmable hardware with LED-embedded fibers and proposing accessible and scalable textile preparation methods. Additionally, our system uses soft textile surfaces that can be integrated into public environments, such as automotive or theater seatings, ensuring greater cleanliness in spaces where soft textiles are still preferred to allow for a more comfortable experience without compromising the cleanliness of the surface.

We use “self-cleaning” to describe the textile’s ability to degrade and inactivate most of the organic contaminants present on the surface of the textile, specifically to remove stains and to inhibit bacterial growth. We designed our system to self-clean by using LED-embedded fibers that activate a photocatalytic reaction on the textile surface pretreated with corona discharge and coated with Titanium Dioxide nanoparticles doped with Nitrogen (TiO_2_/N). The photocatalytic properties of TiO2 have long been known and extensively studied.^4,5^ When exposed to the light emitted by the embedded LEDs, photocatalysis occurs within the nanocoating at the surface of the textile. This results in the breaking down of organic matter into harmless byproducts such as CO_2_ and H_2_O, and the removal of stains and harmful bacteria and viruses from the surface.

Our *self-cleaning textile system* is extending prior work that has explored the cleaning ability of photocatalytic nanoparticles (TiO_2_, ZnO, Fe_2_O_3_, CdS, WO_3_, SnO_2_, or ZnS) on textiles (cotton, nylon, and polyester).^5−9^ So far, TiO_2_ nanoparticles are considered to be cost-efficient, nontoxic, and consequently the most researched photocatalytic nanocoatings for cleaning textiles.^9−19^ Researchers have worked toward narrowing the band gap energy of TiO_2_ by doping the nanoparticles with silver,^20^ manganese,^21^ or nitrogen,^22,23^ to allow for it to be activated by visible light as opposed to UV light. In this work we focused on the scalability and accessibility of our system, thus, rather than developing a lab-based coating, we opted for a commercial product consisting of nitrogen-doped titanium dioxide (TiO_2_/N), namely USA Nanocoat Indoor LumaClean^24^ that has been used by past research to design interactive self-cleaning textiles.^25,26^ In this work, we expand on prior research by introducing a prototype for a self-cleaning textile that can be embedded in high traffic, close contact soft surfaces such as seat covers, and potentially used in cinemas and rideshare vehicles. Our self-cleaning textile leverages state-of-the-art fibers that *embed the illuminating diode fibers internally into the fabric*,^27^ thus eliminating the dependency on external light sources to clean the surface. These fibers allow light from multiple point sources to scatter evenly across the textile surface and thereby facilitate uniform self-cleaning on textiles that have a high risk of stain and bacterial accumulation. The material and structural choices for the self-cleaning system were based on the comparative study we conducted between various methods for pretreatment, coating, and curing. We built the final textile system by stacking the LED fibers, and reflective and diffusive textile layers to enable modularity and facilitate further improvements. Lastly, we evaluated the proposed self-cleaning textile system using stain degradation and bacterial inhibition tests.

In terms of designing our self-cleaning system, we opted for a scalable solution that can be integrated quickly within existing textiles, specifically, a combination of commercially available materials and accessible methods. We performed experiments to study the effectiveness of polyester as a base textile for self-cleaning since polyester is the most widely used commercial textile for our targeted applications (public seating).^28,29^ The experiments consisted of coating polyester with a photocatalytic layer (USA Nanocoat Indoor LumaClean coating^24^) and evaluating its stain degradation properties. We then run comparison experiments to cotton, and nylon, the other two most commonly used organic and synthetic textiles.^30,31^ Our experiments show that polyester can degrade stains within a similar range as cotton and nylon, and thus can be a good candidate for a self-cleaning base textile.

In terms of methods, we focused on accessibility, by exploring methods that can be replicated without the need for highly specialized equipment. We thus investigated a variety of pretreatment methods (no pretreatment, a commercial primer pretreatment, corona pretreatment, and a combination of the commercial primer and corona pretreatments), curing methods (room temperature for 7 days and elevated temperature for 10 min), and coating methods (spraying, rolling, dip coating, and painting). We used Energy-dispersive X-ray spectroscopy (EDS) to analyze the uniformity of the coating on the textiles. On the treated, coated, and cured textiles, we performed another set of experiments to study the stain degradation of coffee, often used as a common staining agent in evaluating self-cleaning textiles,^26,32,33^ and that has potential real-world interaction with the self-cleaning textile.

We engineered our system to be modular, being made of different scalable and reconfigurable layers. The system consists of the polyester layer pretreated and coated with TiO_2_/N nanoparticles that sits on top of a spacer textile, an LED fiber layer, and a reflective base layer. Together, the spacer, LED fiber, and reflective layers evenly disperse light across the entire polyester textile layer for total self-cleaning coverage. In addition to uniform light dispersion, the layering approach is modular, scalable and reconfigurable, thus overcoming the limitations of past work, which weave^26^ and knit^25^ LEDs directly into the textiles coated with TiO_2_/N nanoparticles.

We then evaluated the self-cleaning system in terms of antibacterial properties, quantifying its ability to reduce the growth of Gram-positive and Gram-negative bacteria. To capture the potential impact of any interference from the surrounding lighting, we also tested the system under different ambient lighting conditions. We thus evaluated the stain degradation property of our system in two different environments that emulate real-life situations: little to no ambient light (e.g., nighttime, dark movie theaters) and room light (e.g., daylight, office lighting). The experimental results show that our self-cleaning system is able to degrade the stains and reduce bacterial activity within the range of the AATCC (American Association of Textile Chemists and Colorists) 100–2019 test standard, meaning that our fabric system effectively kills or inhibits bacteria over a 24-h period. The success of our results in our system being able to clean itself indicates promising future applications into hygienic, public soft surfaces.

## Materials and Methods

### Materials

We purchased three types of textiles from JOANN Fabrics and Crafts: Cotton (tabby weave),^34^ Polyester (Casa Collection white polyester satin solids fabric),^35^ and Inherent Flame Retardant (IFR) nylon (ripstop weave).^36^ We chose these three fibers because they are the three most widely used textiles; cotton is the most widely used natural fiber,^30^ while polyester is the most widely used synthetic fiber.^31^

To coat the textiles, we used commercial nitrogen-doped TiO_2_ nanoparticles packaged as the product LumaClean Indoor from USA Nanocoat. This coating is suitable for our testing environments (with low light, and room light conditions) as it extends the absorption frequency into the visible range of light, especially in low ambient lighting conditions with an illuminance of ∼580 lx. For pretreatment, we used LumaClean Primer, from USA Nanocoat,^24^ a nanocoating that helps adhere the corresponding TiO_2_/N coating to surfaces as recommended by USA Nanocoat.

For the stain degradation experiments, we used a solution of 1.0% Medaglia D’Oro Espresso Instant Dark Roast Coffee to 99% deionized water. Lastly, for antibacterial testing, we used *Escherichia coli* and *Staphylococcus aureus* grown in a liquid Luria Broth and Nutrient Broth media respectively, purchased from Carolina Biological.^37^

### Textile Preparation Method

Consistent with the AATCC TM100 standard,^38^ we prepared 5 cm by 5 cm textile samples by washing them in a 40:1 (by weight) liquid-to-textile cleaning solution that consisted of 2g/L of nonionic cleaning agent to distilled water. The textile was sonicated in this cleaning solution at a frequency of 40 kHz and temperature of 60 °C for 15 min. Once washed, we sonicated the textile at a frequency of 40 kHz in room-temperature distilled water for 15 min. We then removed the textile from the sonication bath and dried the textile at room temperature on a sterile flat surface, which took approximately 3 h depending on the type of textile (nylon drying the fastest and cotton drying the slowest).

### Pretreatment Methods

As pretreatment methods, we tested the corona discharge and primer coating, resulting in four experiments that included the negative control and the combination of the two treatments, as follows:

1. No Pretreatment: Control samples were only washed following our textile preparation method before the TiO_2_/N coating was applied.
2. Primer: We applied USA Nanocoat LumaClean Primer to the surface of the textile, which is the recommended pretreatment for the corresponding USA Nanocoat LumaClean coating. This pretreatment is made primarily up of Peroxotitanium acid and is said to increase adhesion and act as a binding agent between the surface and the LumaClean coating. We applied the primer by dipping the 5 cm by 5 cm textile samples in a bath containing 5 mL of primer. The textile samples were soaked in the bath for 5 min and then removed and left to dry at room temperature on a clean flat surface.
3. Corona: Corona pretreatment was applied using a hand-held commercial device (BD-20AC Laboratory Corona Treater, Electro-Technic Products Inc.^39^) at atmospheric pressure. We chose to test corona pretreatment as it has been used to improve the adhesion of dyes and coatings to textiles.^40^ Samples were placed on a clean lab bench and the device was held 2 cm above the textile. The corona plasma was discharged through a 6 cm wide electrode which was moved consistently across the surface of the textile sample. We experimented with a range of voltages (34–50 kV) and times (15–120 s), using dyne testing^41^ to determine the surface energy of the treated samples. By applying corona discharge to the textile, the surface energy and polarity increase, thus increasing the roughness of the fiber surface, which improves the wettability of the textile as a whole.^42^ Therefore, dyne testing gave us an understanding of how long the corona pretreatment lasted on the surface of the nylon and how much it improved the wettability of the surface. Through our preliminary dyne testing, we determined that we would set the corona plasma discharge device to 50 kV and apply the discharge for 120 s as our corona pretreatment method.
4. Corona + Primer: Our final pretreatment method was a combination of the corona and primer pretreatments. We first applied the corona pretreatment to the surface of the textile sample (50 kV for 120 s, 2 cm away from the surface of the textile) and then immediately submerged the textile in the primer pretreatment. After soaking the textile in the primer, we let the samples dry at room temperature.

### Coating Methods

We coated the 5 × 5 cm textile samples via four different accessible and scalable methods as follows:

1. Painting: We painted approximately 5 mL of nanocoating onto the surface of the textile using a natural bristle brush. This method is recommended by the manufacturer, USA Nanocoat.
2. Dip Coating: We dipped textiles in a 5 mL bath of nanocoating, letting the textiles sit in the bath for 5 min before removing them. Immersing was proposed as a coating method by both Zhang et al.^14^ and Li et al.^43^
3. Spraying: We sprayed approximately 5 mL of nanocoating onto the surface of the textile using a 200 mL hand-held spray bottle. This method was adapted from the high-pressure airbrushing method used by Zahid et al.^21^
4. Rolling: We rolled 5 mL nanocoating onto the surface of the textile using a rubber roller used for printmaking to emulate rollers used for dye application in the textile industry.

### Curing Methods

We evaluated two different types of curing methods:

1. Room Temperature: Coated samples were cured on a clean flat surface at room temperature under no light exposure for 7 days. This was the method recommended by the manufacturer USA Nanocoat.
2. Elevated Temperature: Similarly to the method proposed by Emami et al.,^44^ coated samples were dried in a dehydrator oven at a temperature of 70 C for 5 min, and then cured at 90 C for another 5 min.

### Staining Method

Each sample was submerged in 10 mL of a 1.0% coffee solution for 5 min and dried at room temperature in complete darkness. The stained samples were stored in a dark drawer until they were used for the stain degradation tests.

### Light-Integrated Textile System

**Figure 1:**
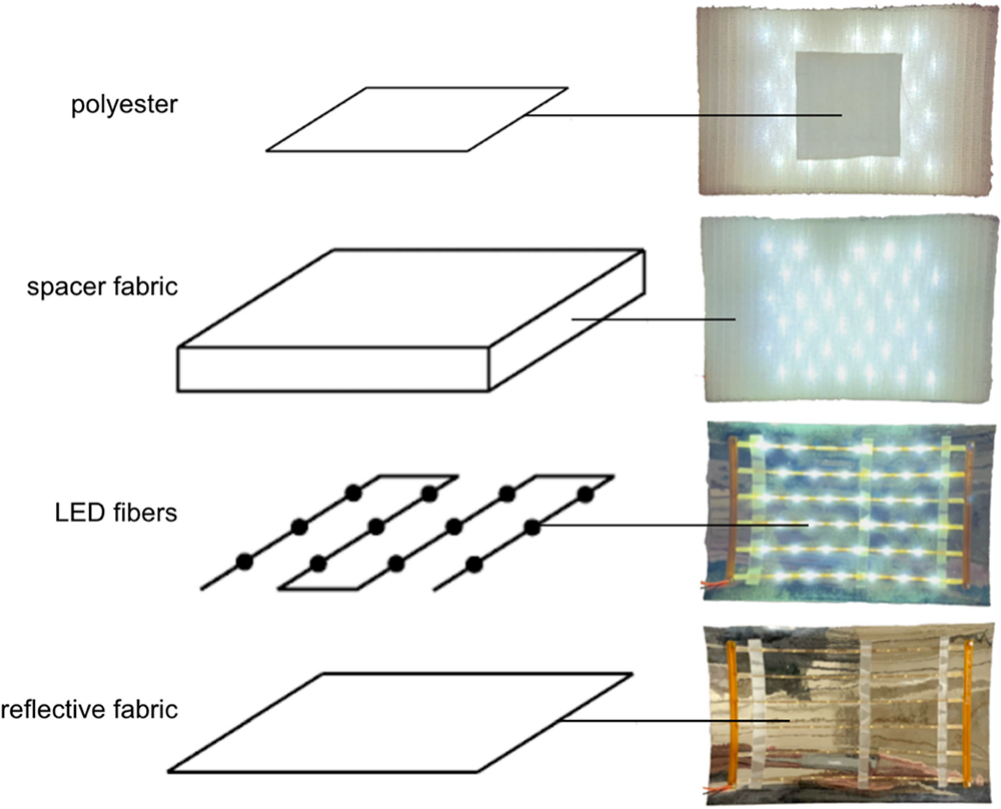
Our proposed light integrated textile system. Left: Schematic representations of the full stack. Right: Pictures of the prototype that stacks a reflective layer, an illumination layer with LED fibers, and a spacer fabric with light diffusion characteristics.

To deliver uniform illumination to the textile surface to activate the self-cleaning of the coated textile, a composite textile stack structure was built as shown in Figure 1. The illuminating layer was constructed by connecting 6 custom-made LED fibers in series. Each LED fiber was made from poly(ether imide) (PEI) (fiber cladding), containing two internal parallel copper wires and 6 LEDs (Genesis WA0606) connected to the wires in parallel. The spacing between LEDs on each fiber was 15 mm. The illumination layer was embedded between a reflective layer (CS Hyde metalized PET film placed at the bottom of the stack) and a translucent spacer fabric (Apex Mills 9 mm high WKS polyester warp knit monofilament spacer fabric, 16.3 oz/yd2). The reflective layer angles the light toward the textile surface and the spacer fabric disperses the light on the textile surface. A coated fabric of interest (polyester swatch) was placed on top of the spacer fabric and its self-cleaning properties were evaluated in this study. We tested three different intensities: high (30 mA = 2840 lx), medium (20 mA = 1260 lx), and low (10 mA = 580 lx). The internal LED light source had a correlated color temperature of 5000 K and was situated inside the textile stack 1 cm from the outer fabric.

The illumination layer in the textile stack was connected to a power supply set at constant current and hovering voltage, which we used to control the intensity of the illumination and make our textile system programmable for applications in theater and automobile seating. To create a demonstrable prototype, we packaged our textile stack within a white polyester seat cover with an antislip textured base to prevent slippage when being sat on (Figure 7).

### Stain Degradation Testing

Stain degradation testing involved staining the polyester swatch with coffee using our staining method and irradiating it with a light source to study the stain degradation activity. The color of the stain was measured over time as it was exposed to light. For measurements, we used the CM-700d Spectrophotometer from Konica Minolta^45^ set at an aperture of 8 mm with an 8° viewing angle and with a D65 CIE Standard Illuminant. We quantified the degree of visible color change in the CIELAB color space, denoted by Δ*E* in eq 1, wherein any value >1 is detectable by the naked eye. In CIELAB, *L* indicates lightness and *a* and *b* are the chromaticity coordinates.
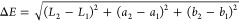
1

**Figure 2:**
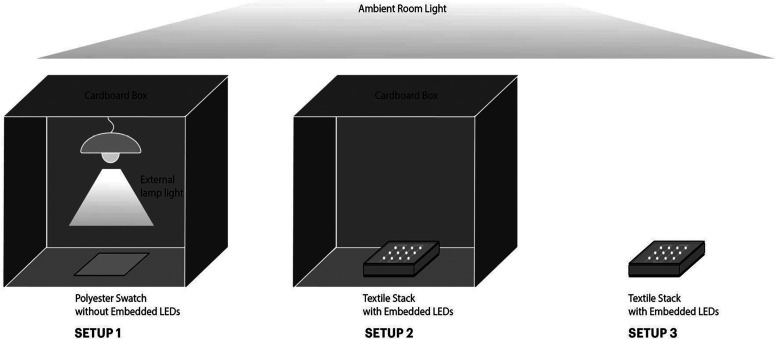
Different setups used for stain degradation testing.

Stain degradation testing was performed under 3 different light setups as shown in Figure 2. Setup 1 involved the use of external light from a table lamp on the polyester swatch. To avoid ambient light interference, we placed this setup underneath a box. Setup 2 consisted of using the LED integrated textile system, also placed underneath a box to shield the system from ambient light interference. Setup 3 involved using the LED integrated textile system, however, this setup was not shielded from ambient light and was exposed to room lighting.

1. External Light Desk Lamp Setup (Setup 1): Stain degradation tests for the initial comparative study for nanocoating optimization were performed with an external light (desk lamp) – the setup most commonly used in prior literature.^46,47^ The stained textile sample was placed on a lab bench at a 32 cm distance from a 12-W eye-caring LED desk lamp from JUKSTG. The lamp emits a correlated color temperature of 5000 K and an illuminance of 2365 lx. The setup was placed in a controlled setting that prevented interference with any other light source, e.g., ambient light.
2. Light Integrated Textile Setup with No Ambient Room Light (Setup 2): Stain degradation tests of the polyester swatch placed on the LED fiber-integrated textile system were conducted using Setup 2. In this setup, the polyester swatch was illuminated using the light from the LED fibers, and no external lamp was used. The setup was shielded from any other light source, e.g., ambient room lighting using a cardboard box.
3. Light Integrated Textile Setup with Ambient Room Light (Setup 3): To understand how stain degradation activity is affected by ambient lighting (room lighting), we used Setup 3. In this setup, the polyester swatch was illuminated using the light from the LEDs, and no external lamp was used. However, the setup was exposed to ambient room lighting.

### EDS Analysis

We coated the textiles with a 5 nm layer of platinum (Pt) with a Cressington 208HR sputter coater to avoid charging during imaging. Samples were then cut into 1.25 cm by 1.25 cm squares and placed in a Hitachi SU3500 SEM operating at 10 kV acceleration voltage. We took EDS measurements of Ti% by weight % at the center and edge of each sample to measure the uniformity of the nanocoating.

### Antimicrobial Textile Testing

**Figure 3:**
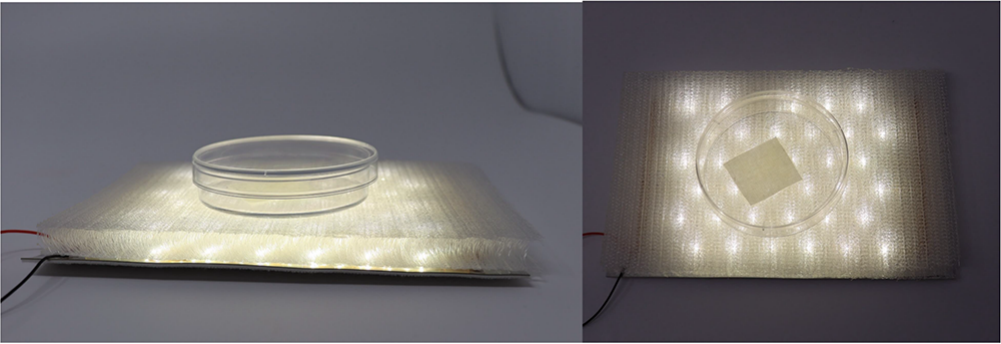
Antimicrobial testing setup over LED fiber-integrated textile stack.

To test the antibacterial properties of our textile we used the textile industry’s standard AATCC Test Method 100–2019 developed by the American Association of Textile Chemists and Colorists to assess the antibacterial finish on textile materials.^38^ We used the Shake Flask Method to test bacterial activity on the selected textile: polyester treated with corona discharge and primer, and coated by dipping. To fit in the flasks and Petri dishes, the 5 cm by 5 cm coated textile swatches were cut into 2.5 × 2.5 cm squares. We then inoculated them with 250 μL of target bacteria, and then exposed them to the light-integrated textile system for 2 h in a Petri dish sealed with parafilm to avoid evaporation (Figure 3). The light exposure setup is built to mimic how the textile would sit on the LEDs in a real-world scenario. After light exposure, the swatches were placed into flasks of 50 mL neutralizing broth (LB medium). We vigorously shook each flask for approximately 2 min, then inoculated 6 cm agar plates with 30 μL of the medium. Serial dilutions were made, and the plates were incubated overnight at 37 C. After incubation, colonies of recovered bacteria were counted and used to determine percent reductions. We used this protocol to test for antibacterial reduction of Gram-negative bacteria *Escherichia coli*(*E. coli*) K12, and Gram-positive bacteria*Staphylococcus aureus*.

## Results and Discussion

### Stain Degradation Testing with External Light Setup (Setup 1)

Using the external light (Setup 1 in Figure 2), we ran experiments to test the stain degradation capabilities of polyester in comparison to cotton, and nylon, the most commonly used and commercially available organic and synthetic textiles, respectively.^30,31^ The results presented in Table 1 indicate that the cotton-based textile system degrades coffee the most, followed by polyester and then nylon. We selected polyester as textile cover—the most widely material in the envisioned applications for public seating (theaters, shared transportation, etc.). Selection criteria included material availability (to ensure the scalability of the system) and comfort (to ensure the usability of the system).

**Table 1:** Comparison of Δ*E* values after 24 h for Different Textiles, Textile Coating Methods, Pretreatment Methods, and Curing Conditions for Stain Degradation^a^

textile	pretreatment method	curing method	coating method	Δ*E* after 24 h
cotton	none	*RT* for 7 days	painting	12.02 ± 0.32
polyester				7.65 ± 0.42
nylon				3.80 ± 0.20
polyester	primer	*RT* for 7 days	painting	8.10 ± 0.30
	corona			8.58 ± 0.18
	corona + primer			9.11 ± 0.69
polyester	none	elevated	painting	2.93 ± 0.30
	corona + primer			3.95 ± 0.34
polyester	none	*RT* for 7 days	spraying	5.00 ± 0.27
	none		rolling	5.88 ± 0.70
	none		dipping	8.37 ± 0.12
polyester	corona + primer	*RT* for 7 days	dipping	16.44 ± 0.19

a The combination of polyester pretreated with corona + primer via the dipping method and cured at *RT* for 7 days resulted in the most effective stain degradation (Δ*E* = 16.44 ± 0.19).

The next set of experiments were designed to determine if the polyester’s effectiveness in degrading stains could be improved upon pretreatment with corona and primer. After pretreatment, we coated and cured the polyester swatches consistent to the previous experiment. The results in Table 1 indicate that pretreating polyester with both corona and primer is most effective at stain degradation. This is consistent with our understanding that corona pretreatment improves the adhesion of the primer to the textile, which further improves the uniformity and adhesion of the TiO_2_ nanocoating to the textile cover.

Next, we investigated the impact of curing temperature on the polyester’s effectiveness for stain degradation. We compare curing the coated polyester textile at RT for 7 days (recommended by the commercial manufacturer LumaClean) with curing at 70 C for 5 min followed by 90 C for another 5 min (method proposed by Emami et al.^44^). The results in Table 1 indicate that RT curing resulted in more destaining than heated curing both with and without any corona + primer pretreatment. While heat has been found to improve the curing of nanoparticles on textiles,^47^ we hypothesize that in our case, the rate of heating was too rapid, potentially altering the effectiveness of the photocatalytic nanoparticles.

We then compared our four coating methods: painting, dipping, spraying, and rolling. For this test, we used our best-so-far curing method (RT for 7 days), and no pretreatment to speed up the experiments. The results in Table 1 indicate that the dipping method resulted in the most destaining, followed by painting, rolling, and, last, spraying. Last, we compared the coating methods with the best results (dipping and painting) under treatment with corona and primer. The results in Table 1 indicate that the corona + primer pretreatment combined with the dipping coating method and RT curing resulted in polyester samples with the greatest stain degradation capability. We will call this combination our “optimized” or “best performing” textile cover.

### EDS Testing

**Figure 4:**
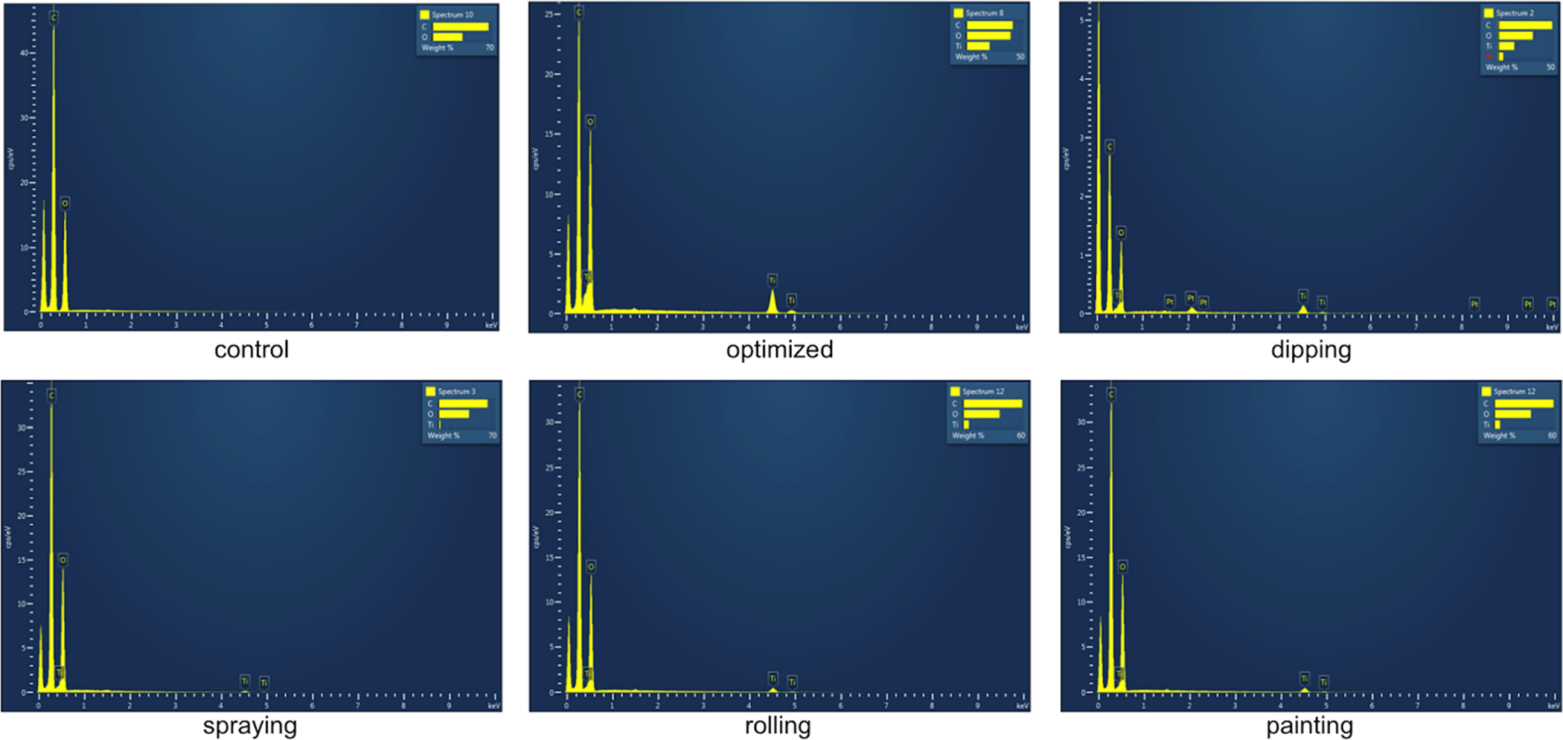
EDS spectra of the textile via different coating methods and with the optimized coating method (cured at room temperature).

We used EDS to quantify the percentage of titanium (Ti%) by weight as an indicator for the amount of TiO_2_ nanoparticles on the treated textile. We used an EDS spot size of 775 μm by 525 μm and took a measurement in the middle and the edge of the 1.25 × 1.25 textiles to gain an understanding of the Ti coverage across the textile. The percentage of Ti provides a good indication of how much nanocoating is adhered to the surface of the textile. Uniformity was calculated as the ratio of Ti% at the edge to Ti% at the middle, expressed as a percentage, where values closer to 100% indicate a more even distribution. As seen in Table 2 and Figure 4, the optimized swatch, (i.e., the polyester pretreated with corona + primer, then coated via dipping, and cured at room temperature for 7 days) has the highest percentage of titanium (Ti) on the surface of the textile. Uniformity analysis further confirmed that this optimized method achieved the most even Ti distribution, with a uniformity of 72.06%, compared to lower uniformity observed for spraying (54.08%) and rolling (58.78%). However, we note that the primer also contains Ti, which can also impact these results. Based on our initial stain degradation tests in combination with these EDS results, the following experiments (stain degradation on the light integrated system (using setups 2 and 3 and antibacterial testing) all utilize our most optimal textile cover.

**Table 2:** Comparison of the Amount of Titanium on the Surface of the Textile via Different Coating Methods and with the Optimized Coating Method (Cured at Room Temperature)

	Ti % (middle of the sample)	Ti % (edge of the sample)	uniformity (%)
control (untreated, uncoated)	0.00	0.00	N/A
spraying (untreated)	4.66	2.52	54.08
rolling (untreated)	8.49	4.99	58.78
painting (untreated)	9.37	5.03	53.68
dipping (untreated)	14.88	8.97	60.28
best-performing textile cover	35.23	25.39	72.06

### Stain Degradation Testing with Light-Integrated Textile System with No Ambient Light (Setup 2)

As shown in Figure 1, our proposed system integrates LED fibers within a composite textile stack structure, covered with the treated polyester textile. For this set of experiments, we integrated our best-performing textile cover with the proposed system as shown in the Setup 2 in Figure 2 and compared the results with those under external light (Setup 1). The results in Table 3 indicate that our proposed textile system with integrated LEDs performs better in terms of destaining the optimized textile cover as compared to the external light source. This is likely because of the proximity of the integrated LEDs to the textile cover and the higher luminous flux of the LEDs (1 cm away at 2840 lx) as compared to the external light setup (32 cm away at 2365 lx). The two sources were roughly equal in intensity at source but having the LEDs closer to the surface of the textile cover increases the lux values at the point of absorption on the textile cover surface. We note that the textile is optimally pretreated and coated the same on both sides, so the directionality of the light (i.e., the external light hitting the top of the textile versus the integrated LEDs shining through the bottom of the textile) should not make a difference. Moreover, the LEDs integrated within the system are comparable to the LEDs within the external lamp; both the LED-integrated system and the external lamp have a correlated color temperature of 5000 K.

**Table 3:** Comparison of External Light Setup (Setup 1) and Light-Integrated Textile System Setup (Setup 2) with the Optimally Treated Polyester Cover^a^

textile	light source	Δ*E* after 24 h
best-performing textile cover	external light (setup 1 in Figure 2)	16.44 ± 0.19
	integrated LED (setup 2 in Figure 2)	22.69 ± 0.3

a We took *N* = 3 measurements per textile sample at the moment of staining and after 24 h of light exposure to determine average stain degradation and deviation (Δ*E*).

### Stain Degradation Testing with Light-Integrated Textile System with Ambient Light (Setup 3)

**Figure 5:**
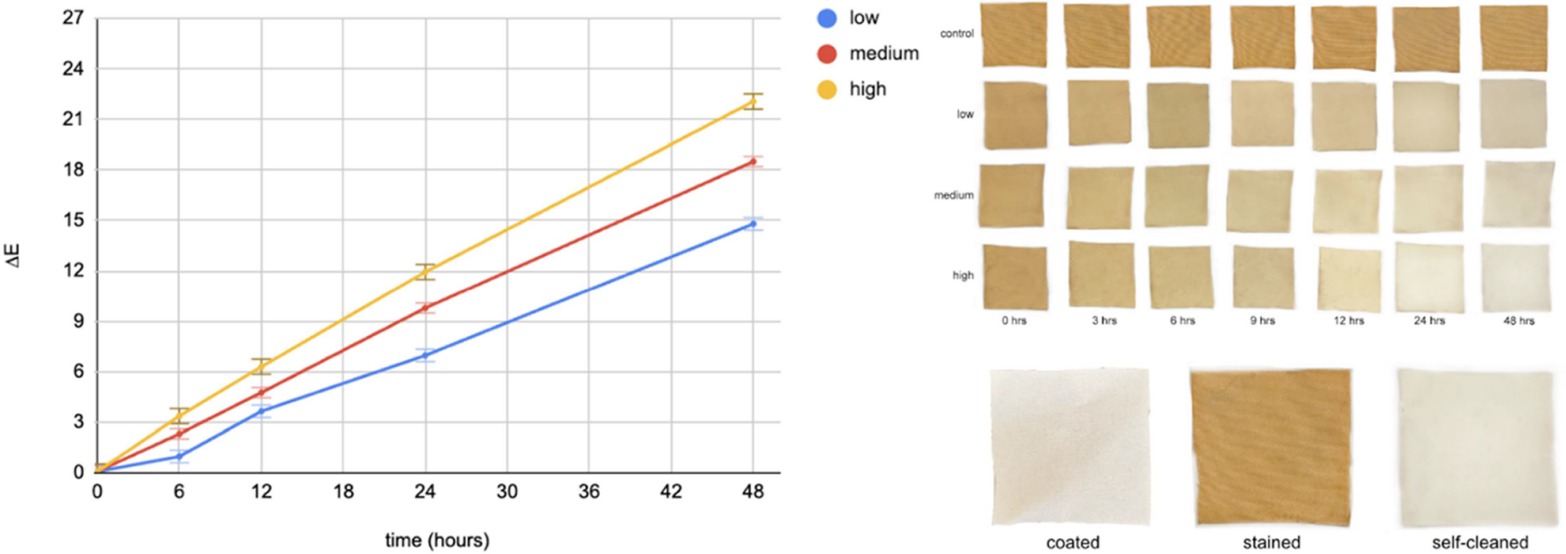
Night-time simulation: stain degradation of optimal textile with the integrated light system at three different intensities over 48 h without ambient light (setup 2).

**Figure 6:**
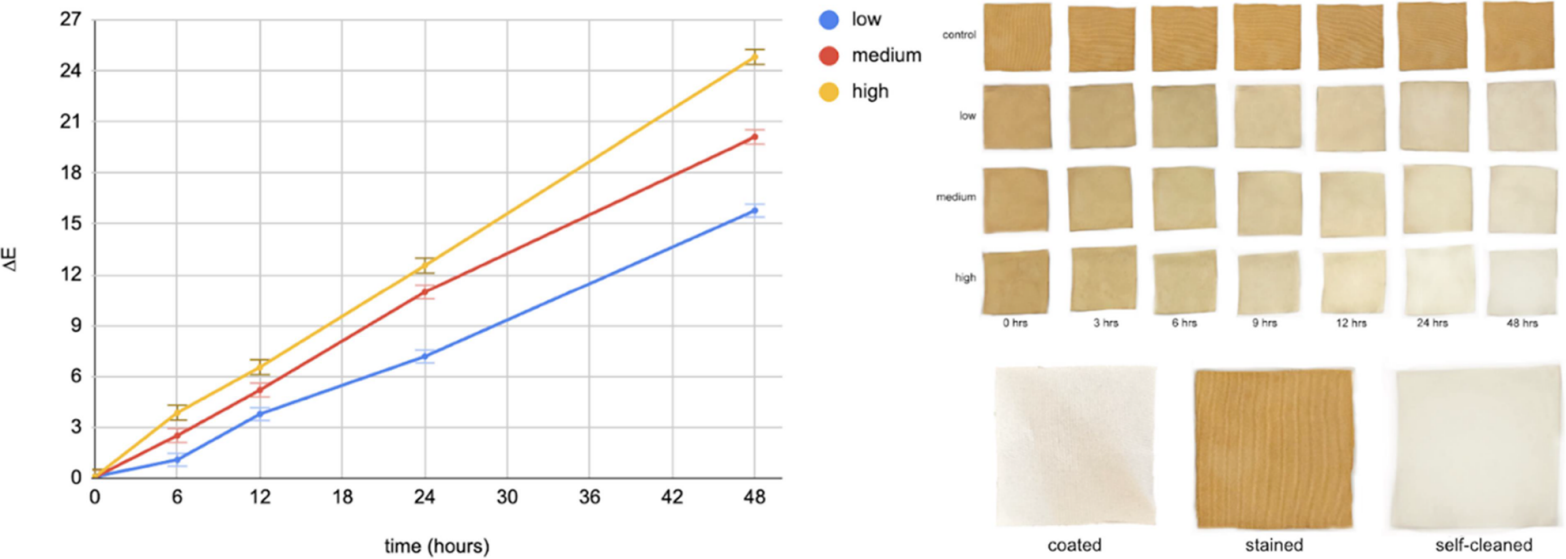
Daytime simulation: stain degradation of optimal textile with the integrated light system at three different intensities over 48 h in the presence of ambient light (setup 3).

So far, our experiments assumed controlled light settings (Setups 1 and 2), and in real-life this scenario is mostly possible during nighttime. During the day, our system will encounter “ambient” light - generated by unrelated sources (e.g., the bulbs in the room), and usually occurring transiently in the environment. To investigate if the ambient light can aid our system significantly, we simulated day conditions by turning on the overhead fluorescent room lighting as shown in Setup 3 in Figure 2. We set our LED integrated textile system to illuminate (internally, from the embedded LEDs) at low (10 mA = 580 lx), medium (20 mA = 1260 lx), and high (30 mA = 2840 lx) intensities. Figures 5 and 6 show the stain degradation (Δ*E* values and qualitative pictures). Our assumption was that the ambient light would impact significantly the stain degradation process, however we did not notice significant differences after 24 h. We chose to continue the experiment for another day, reporting the results over 48 h. While the ambient light does not aid significantly in stain degradation even after 48 h exposure, the results indicate the increasing the LED-light intensity and the duration of exposure results in more effective stain degradation. This shows that our light-integrated self-cleaning system performs very similarly in both day and night conditions, and can be used in indoor applications, even overnight, without any significant decrease in cleaning ability.

### Testing the Antimicrobial Properties

Our antimicrobial testing was performed per the AATCC Test Method 100–2019. Our testing consisted of sample preparation, inoculation, incubation, washing/shaking out, and counting. We performed the tests using the staple organisms for Gram-negative and Gram-positive bacteria, which are *Escherichia coli* (*E. coli*) and *Staphylococcus aureus* (*S. aureus*), respectively. The results for *E. coli*, show 71% fewer colonies counted on the treated sample than on the untreated sample, after 2 h of exposure to the proposed light-integrated system. The follow-up dilutions show an 84% decrease at 10^1^. We obtained a significant cease of activity at 10^2^ dilution (less than one colony at 10^2^), which corresponds to a percent reduction of 99.97%. The results for *S. aureus*, show 54% fewer colonies counted on the treated sample than on the untreated sample, after 2 h of exposure to the light-integrated system. The follow-up dilutions show a 66% decrease at 10^–1^, and a 99.93% decrease at 10^–2^ dilution, slightly less effective than for *E. coli*. After 8 h of exposure to the light-integrated system, we observed a similar percentage reduction of 99.96% for *S. aureus*.

These results presented in Table 4 align our LED-integrated system with the standard testing of other self-cleaning textiles.^48−50^ Thus, we show that the proposed system can significantly inhibit the microbial activity of *E. coli* and *S. aureus*, commonly occurring bacteria on public surfaces, in under 8 h of exposure time.

**Table 4:** Antimicrobial Testing^a^

textile	bacteria	exposure time (h)	growth reduction (%)
best-performing textile cover	*E. coli*	2	99.97
	*S. aureus*	2	99.93
		8	99.96

a Using the AATCC Test Method 100-2019, we tested the antimicrobial properties of the optimal textile under our proposed LED system without ambient light (setup 2). The tests for *Escherichia coli* (*E. coli*) and *Staphylococcus aureus* (*S. aureus*) show over 95% microbial activity inhibition after 2 h of exposure.

### Final Prototype and Mechanism of Action

**Figure 7:**
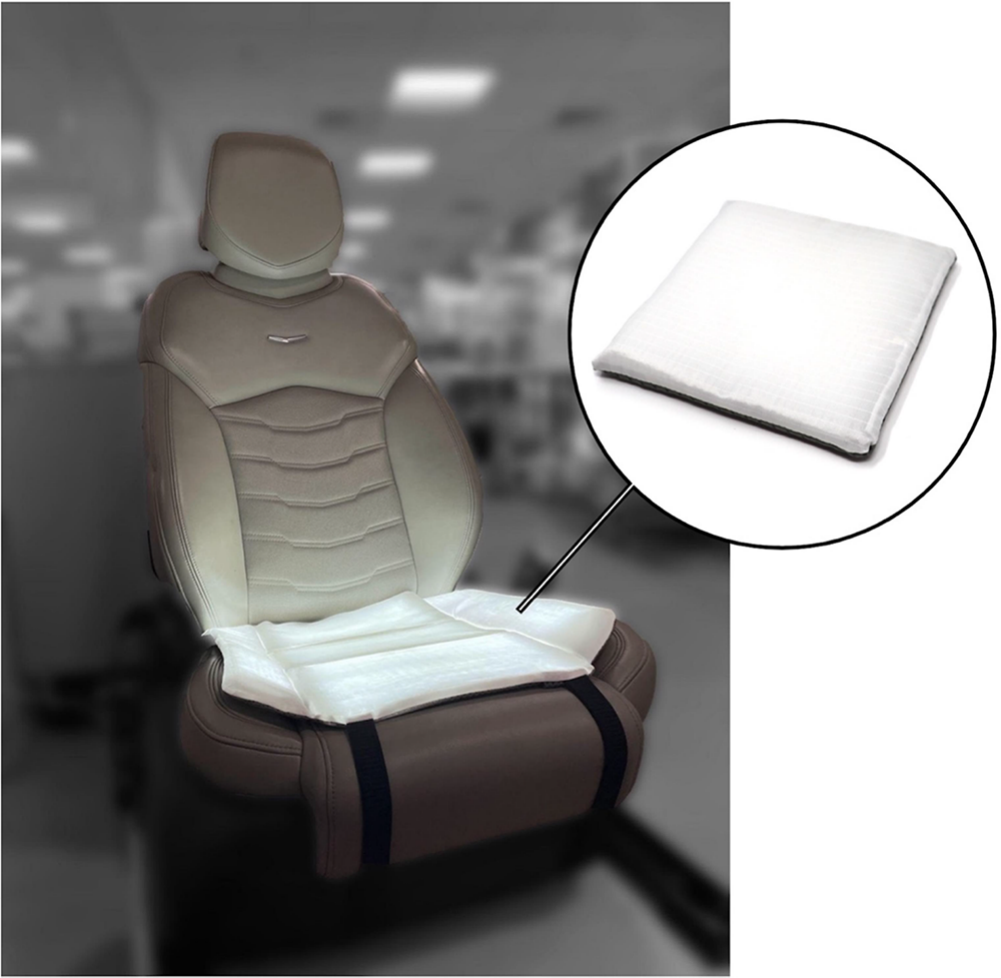
Prototype: Our proposed LED integrated textile system is further packaged within a white polyester seat cover with an antislip textured base. We envision the seat cover to be programmable for periodic self-cleaning and for real-time response to the user’s needs.

Following the experimental evaluation of our proposed system, we developed a functional prototype for real-world applications (Figure 7). The mechanism of action of the system combines photocatalytic activity with advanced programmability. The nitrogen-doped titanium dioxide (TiO_2_/N) coating on the textile surface is activated by light emitted from the embedded LEDs. When illuminated, the TiO_2_/N absorbs photons, exciting electrons and creating positively charged holes. This generates reactive oxygen species (ROS), such as hydroxyl radicals (·OH) and superoxide anions (O_2_^–^·), which actively degrade organic contaminants into harmless byproducts like carbon dioxide (CO_2_) and water (H_2_O). Additionally, these ROS disrupt bacterial cell walls, effectively inhibiting microbial growth. A key feature of the system is its **programmability**: the intensity of the LED light can be controlled via remote software based on usage requirements, and the lighting system can be automated to turn on and off at specific intervals for self-cleaning. Further enhancements include the integration of embedded sensors into the seat cover, which can detect user activity and activate the self-cleaning system immediately after use. This automation ensures efficient energy usage while maintaining hygiene standards over time.

## Limitations and Future Work

While our LED-integrated textile system demonstrates strong stain degradation and antibacterial characteristics, it requires approximately 48 h to visibly destain the textile and around 8 h to effectively eliminate germs and microbial growth from the surface. This is in contrast to the more immediate results that techniques like washing or scrubbing with a disinfectant might enable. However, it should be noted that washing and scrubbing textiles is not instantaneous either and requires significant time and effort, which can disrupt continuous operation. Running our system continuously or for prolonged periods can keep the buildup of germs and stains to a minimum, ensuring an always-clean surface. Furthermore, our system eliminates the need for manual labor associated with washing or scrubbing, reducing wear and tear, and increasing the longevity of textile surfaces, as well as it can also be incorporated on surfaces that might otherwise never be cleaned.

Due to the nature of use, abrasion is a key concern for our self-cleaning system, as repeated sit/stand activities and friction against users’ clothing can wear down the TiO_2_ coating over time. Anticipating this issue, we specifically selected *LumaClean* (USA Nanocoat) coating for its strong adhesion, high curing strength, and durability. While *LumaClean* demonstrates robustness and recommends reapplication only every 10–15 years, wear and tear may still occur with prolonged use. To further reduce the need for frequent reapplication and extend the coating’s lifespan, hydrophobic laminates offer a promising solution.^51−53^ These laminates provide an additional layer of hydrophobicity and antibacterial protection, while simultaneously acting as a physical barrier to minimize wear and prevent stains.

Our self-cleaning system employs LEDs in the visible spectrum that do not harm human cells (skin and eyes), unlike UV light-based cleaning which, although instantaneous, is harmful and requires specific protocols and the absence of human users. These advantages may outweigh the limitations and suggest that our self-cleaning textile system could be used more frequently, possibly in addition to occasional washing and scrubbing, rather than as a replacement. Lastly, based on the manufacturer’s instructions, the current TiO_2_ nanocoating requires reapplication every 6 months or more for effective cleaning. Our future efforts will focus on characterizing the performance of our system over multiple cleaning and staining cycles and exploring ways to extend the life of the nanocoating to reduce the frequency of reapplication.

## Conclusions

In this work, we outline the development of a modular and accessible, light-embedded self-cleaning textile cover that effectively destains coffee and other similar organic stains and kills surface-borne bacteria even in low external light conditions. To optimize our self-cleaning textile, we conducted a systematic study comparing different commercial textiles, pretreatment methods, coating application methods, and curing methods. Stain degradation experiments and EDS analysis showed that polyester textile pretreated with corona treatment at 50 kV for 120 s followed by a layer of Ti primer, coated with commercial nitrogen-doped TiO_2_ using dip coating, and cured at room temperature for 7 days had the highest self-cleaning capability under external light. Using this textile cover, a textile system was designed by integrating LEDs directly into the textile and adding spacer and reflective textiles to add cushioning and light diffusion properties to the system. Stain degradation testing under varying external lighting conditions showed that in both low light and room light conditions, stain degradation increased linearly with LED intensity and exposure time, with nearly all of the stain gone within 48 h. Antibacterial testing with Gram-negative and Gram-positive bacteria demonstrated bactericidal action with 99.9% efficacy in under 2 h. Our system thus has the potential to be integrated into smart textile interfaces that can be programmed to self-clean automatically based on frequency of use.
